# Ursolic Acid Inhibits Breast Cancer Metastasis by Suppressing Glycolytic Metabolism *via* Activating SP1/Caveolin-1 Signaling

**DOI:** 10.3389/fonc.2021.745584

**Published:** 2021-09-08

**Authors:** Shengqi Wang, Xu Chang, Juping Zhang, Jing Li, Neng Wang, Bowen Yang, Bo Pan, Yifeng Zheng, Xuan Wang, Hesheng Ou, Zhiyu Wang

**Affiliations:** ^1^Section of Science and Technology, Guangxi International Zhuang Medicine Hospital, Guangxi University of Chinese Medicine, Nanning, China; ^2^Guangdong Provincial Key Laboratory of Clinical Research on Traditional Chinese Medicine Syndrome, Guangdong Provincial Academy of Chinese Medical Sciences, Guangdong Provincial Hospital of Chinese Medicine, Guangzhou University of Chinese Medicine, Guangzhou, Guangdong, China; ^3^Department of Mammary Disease, Panyu Hospital of Chinese Medicine, Guangzhou, China; ^4^The Research Center of Integrative Cancer Medicine, Discipline of Integrated Chinese and Western Medicine, The Second Clinical College of Guangzhou University of Chinese Medicine, Guangzhou, China; ^5^Guangdong-Hong Kong-Macau Joint Lab on Chinese Medicine and Immune Disease Research, Guangzhou University of Chinese Medicine, Guangzhou, China; ^6^State Key Laboratory of Dampness Syndrome of Chinese Medicine, The Second Affiliated Hospital of Guangzhou University of Chinese Medicine, Guangzhou, China; ^7^The Research Center for Integrative Medicine, School of Basic Medical Sciences, Guangzhou University of Chinese Medicine, Guangzhou, China

**Keywords:** ursolic acid, breast cancer metastasis, glycolytic metabolism, Caveolin-1, SP1, *Oldenlandia diffusa*

## Abstract

Breast cancer remains the most common malignancy and the leading causality of cancer-associated mortality among women worldwide. With proven efficacy, *Oldenlandia diffusa* has been extensively applied in breast cancer treatment in Traditional Chinese Medicine (TCM) for thousands of years. However, the bioactive compounds of *Oldenlandia diffusa* accounting for its anti-breast cancer activity and the underlying biological mechanisms remain to be uncovered. Herein, bioactivity-guided fractionation suggested ursolic acid as the strongest anti-breast cancer compound in *Oldenlandia diffusa*. Ursolic acid treatment dramatically suppressed the proliferation and promoted mitochondrial-mediated apoptosis in breast cancer cells while brought little cytotoxicities in nonmalignant mammary epithelial cells *in vitro*. Meanwhile, ursolic acid dramatically impaired both the glycolytic metabolism and mitochondrial respiration function of breast cancer cells. Further investigations demonstrated that ursolic acid may impair the glycolytic metabolism of breast cancer cells by activating Caveolin-1 (Cav-1) signaling, as Cav-1 knockdown could partially abrogate the suppressive effect of ursolic acid on that. Mechanistically, ursolic acid could activate SP1-mediated *CAV1* transcription by promoting SP1 expression as well as its binding with *CAV1* promoter region. More meaningfully, ursolic acid administration could dramatically suppress the growth and metastasis of breast cancer in both the zebrafish and mouse xenotransplantation models of breast cancer *in vivo* without any detectable hepatotoxicity, nephrotoxicity or hematotoxicity. This study not only provides preclinical evidence supporting the application of ursolic acid as a promising candidate drug for breast cancer treatment but also sheds novel light on Cav-1 as a druggable target for glycolytic modulation of breast cancer.

## Introduction

Although remarkable advancements have been made in drug development and prognosis improvement of breast cancer (1, 2), it still represents the leading reason for cancer morbidity and mortality among females worldwide (3). The latest global cancer epidemiological data estimated that more than 2.3 million new cases and 685,000 deaths of breast cancer occurred in 2020 worldwide, accounting for 24.5% of newly confirmed cases and 15.5% of death cases among women (3). More seriously, breast cancer incidence is still increasing while its onset age is tending younger globally (4). Breast cancer has become a serious global public health burden. Thus, it is necessary and imperative to discover novel breast cancer therapeutics with high efficiency, low toxicity and low cost to improve the survival of breast cancer patients.

Despite the diversified approaches to anticancer drug development, herbal compounds still play a crucial role in the development of anticancer drugs (5). With reliable efficacies and low toxicities, TCM herbs have been widely applied to treat cancer in China and Asian counties with a long history (6). Accumulating evidence has indicated that TCM herbs were efficient in improving the survival and decreasing the death risks of cancer patients (7, 8). Notably, TCM herbs have been practically appreciated as an important reservoir for anti-cancer drug discovery (9). For example, numerous chemotherapeutics, such as paclitaxel, camptothecin, vincristine and podophyllotoxin, were all developed from phytochemicals. It has been reported that more than 3000 kinds of herbs were used to treat cancer in TCM (9) while *Oldenlandia diffusa* represents one of the most commonly prescribed anti-cancer herbs in TCM (6). It has been reported that *Oldenlandia diffusa* was used as the core medicinal herb for breast cancer treatment in 41.9% of TCM prescriptions in Taiwan (10). Both preclinical studies and clinical trials have convinced the efficacy and safety of *Oldenlandia diffusa* in treating various cancers including breast cancer (11–14). Our previous study also demonstrated that *Oldenlandia diffusa* extract could strongly suppress breast cancer growth and metastasis (15). However, the bioactive compounds of *Oldenlandia diffusa* accounting for the anti-breast cancer activity and its underlying biological mechanisms are still elusive. Given the extensive use of *Oldenlandia diffusa* in the clinic, it is of great clinical significance to elucidate the material basis and action mechanism of *Oldenlandia diffusa* against breast cancer, which can provide a lead compound for novel drug development.

Metabolic abnormality is one of the hallmarks of cancer (16). Therefore, targeting the metabolic differences between normal cells and cancer cells represents a potential cancer treatment strategy (17). Cancer cells predominantly rely on glycolytic metabolism as the primary way to satisfy bioenergetic demands, while normal cells mainly utilize mitochondrial oxidative phosphorylation (OXPHOS) (18). This phenomenon has been named as “Warburg” effect. Breast cancer cells also exhibited elevated glycolytic metabolism when compared with mammary epithelial cells (18). Therefore, pharmacological inhibition of glycolytic metabolism of breast cancer cells represents a promising cancer-selective killing strategy. Nowadays, growing efforts are being made to screen glycolytic inhibitors focusing on the targets in glycolytic pathways. These targets include glucose transporters (GLUTs) and the rate-limiting enzymes of glycolytic metabolism (e.g. hexokinase 2, pyruvate kinase M2, and phosphofructokinase 1). Multiple glycolytic inhibitors have been identified and proven effective in delaying the growth of breast cancer. For example, hexokinase inhibitors 3-bromopyruvate (3-BrPA) and 2-deoxyglucose (2-DG) represent the most extensively used glycolytic inhibitors and have shown encouraging antitumor efficacies in breast cancer (19). However, at present, the existing glycolytic inhibitors have not achieved satisfying clinical outcomes in clinical trials mainly because of their low selectivities and systemic toxicities. One approach to solve this problem is to screen glycolytic inhibitors by focusing on the glycolytic metabolism-related targets whose expressions are not fatal to organisms. Cav-1 is a constituent protein of plasma membrane and has been reported to be closely implicated in cancer tumorigenesis and metabolism modulation (20, 21). Additionally, Cav-1 exhibited decreased expression in breast cancer cells than normal breast epithelial cells, and its overexpression could inhibit the glycolytic metabolism of breast cancer (18, 22). Additionally, Cav-1 overexpression in mice by genetic or pharmacological approaches brought little serious side effects in terms of growth pattern, body weight, developmental and reproductive activities when compared with the wild-type mice (22, 23). These findings suggested Cav-1 as an ideal target for glycolytic inhibitor identification. Until now, few studies have developed glycolytic inhibitors under the bioactivity guidance of Cav-1. Therefore, this study was designed to determine the potential compound targeting Cav-1 expression in *Oldenlandia diffusa* and to explore its pharmacological mechanisms against breast cancer.

In this study, we systematically demonstrated that ursolic acid, the bioactive compound of *Oldenlandia diffusa*, could dramatically suppress breast cancer growth and metastasis by impairing glycolytic metabolism *via* activating SP1/Cav-1 pathway. This study not only highlights ursolic acid as a promising candidate drug for breast cancer treatment but also sheds new insights on Cav-1 as a druggable target for glycolytic modulation of breast cancer.

## Materials and Methods

### Bioactivity-Guided Fractionation and Identification of the Bioactive Compound in *Oldenlandia diffusa*

Firstly, 2.5 kg *Oldenlandia diffusa* was extracted with 95% ethanol for 3 times by refluxing extraction method. After freeze-drying, the 95% ethanol extracts were subjected to successive extraction using solvents of ascending polarity. Subsequently, the above extracts were vacuum dried and screened by comparing their anti-proliferation effects on breast cancer cells using the CCK-8 method. Since the petroleum ether phase extracts exhibited the strongest anti-proliferative effects on breast cancer cells, they were further fractionated by silica-gel column chromatography and eluted with petroleum ether and ethyl acetate. A total of 20 fractions were collected and then subjected to mass spectrometry analysis. The top 10 most abundant compounds were identified and quantified.

### Cell Culture and Transfection

MCF-7, MDA-MB-231, 4T1 and HBL-100 cells were purchased from the American Type Culture Collection. 4T1-Luc cells were obtained by transfecting the luciferase lentiviral vector plasmid into 4T1 cells. Therefore, 4T1-Luc cells fluoresced in the presence of D-luciferin substrate. All cell lines were used between passage numbers 10~20. Cells were cultured in DMEM or RPMI-1640 mediums supplemented with 10% fetal bovine serum. Cells were cultured at 37°C in a humidified incubator containing 5% CO2.

### siRNA Transfection

The commercialized siRNAs for *CAV1* and *SP1* were purchased from Vigene Biosciences and transfected into breast cancer cells using X-tremeGENE siRNA transfection reagent (Cat No. 4476093001, Roche Diagnostics).

### Cell Proliferation and Colony Formation Assay

The cytotoxicities of the tested extracts or compounds in breast cancer cells or mammary epithelial cells were detected using the CCK8 Kit (C0038, Beyotime Biotechnology). Briefly, cells were seeded into the 96-well plate (5×10^3^ cells/per well). After attachment, cells were treated with the extracts of *Oldenlandia diffusa* or the indicated compounds for 24~72 h. After treatment, 20 μl CCK8 was added to each well, and cells were incubated for 2 h. The absorbance of each well was measured at 450 nm using a multifunctional microplate reader. To investigate the long-period inhibitory effect of ursolic acid on breast cancer cell growth, the colony formation assay was conducted as previously reported (15). Briefly, breast cancer cells were seeded in a 6-well plate (500 cells per well). After attachment, cells were treated with 1~30 μM ursolic acid for 6 h and then continued to culture for 10 days. The resultant colonies were fixed with 4% paraformaldehyde and then stained with coomassie blue.

### Cell Cycle Distribution and Apoptosis Detection

Breast cancer cells were treated with ursolic acid (0~30 μM) for 48 h. Cell cycle distribution was detected using the Cell Cycle Detection Kit (KGA512, KeyGEN BioTECH). Cell apoptosis was detected using Annexin V-FITC/PI Apoptosis Detection Kit (KGA107, KeyGEN BioTECH). Briefly, breast cancer cells were seeded into 6-well plates (5×10^5^ cells per well). After treatment with 1~30 μM ursolic acid for 48 h, cells were stained with Annexin V-FITC (5 μl/test) and PI (10 μl/test) for 5 mins at room temperature. Flow cytometry was conducted to measure the cell cycle distribution and apoptosis of breast cancer cells using the NovoCyte flow cytometer (ACEA Biosciences).

### Mitochondrial Membrane Potential Detection

Mitochondrial membrane potential detection assay was conducted to investigate the effect of ursolic acid on the mitochondrial membrane potential and apoptosis of breast cancer cells. Briefly, breast cancer cells were treated with ursolic acid (0~30 μM) for 48 h. Then, cells were collected and subjected to JC-1 probe staining assay using the JC-1 Probe Kit (Beyotime, C2006). JC-1 green fluorescence intensities of breast cancer cells were analyzed using the NovoCyte flow cytometer.

### Western Blotting and Immunofluorescence Assay

Western blotting assay and immunofluorescence assay were performed as previously reported (18). Briefly, total protein extracts were prepared by lysing cells or tumor tissues using RIPA (P0013B, Beyotime). Protein concentrations were quantified using the BCA method. Equal amounts of protein (30 μg) were resolved by SDS-PAGE. Densitometric analyses of protein bands were performed using the Gel-Pro analyzer 4 software. For tissue immunofluorescence assay, the tissue sections were dewaxed, hydrated, and subjected to antigen retrieval by incubating slides in the boiled citrate buffer (0.01 M, pH 6.0). For cell immunofluorescence assay, breast cancer cells were cultured in the confocal dish, treated with 10~20 μM ursolic acid for 48 h, and fixed with 4% paraformaldehyde for 20 mins. Then, the tissue sections or cells were permeabilized with 0.25% Triton X-100 for 20 mins. After blocking, the tissue sections or cells were incubated with primary antibodies overnight, followed by incubation with the secondary antibodies. DAPI was used to visualize the nuclei. Fluorescence images were photographed using the LSM710 confocal microscope (Zeiss, Jena, Germany). The following antibodies were used including Cav-1 antibody (3238, Cell Signaling Technology, CST), β-actin antibody (4970S, CST), Bcl2 antibody (12789-1-AP, Proteintech), Bax antibody (50599-2-Ig, Proteintech), PARP antibody (13371-1-AP, Proteintech), PGC-1α antibody (13067, Santa Cruz), Nrf-1 antibody (sc-13031, Santa Cruz), LDH-A antibody (3582S, CST), c-Myc antibody (5605, CST), SP1 antibody (21962-1-AP, Proteintech), LDHA antibody (66287-1-Ig, Proteintech), Alexa Fluor^®^ 555 conjugated-anti-rabbit IgG (4413S, CST) and Alexa Fluor^®^ 488 conjugated-anti-mouse IgG (4408S, CST).

### Lactate Concentration Detection Assay

The effect of ursolic acid on lactate production of breast cancer cells was investigated as we previously reported (18). Briefly, breast cancer cells were treated with 1~30 μM ursolic acid or 100 μM 3-BrPA for 48 h. After treatment, 1×10^6^ breast cancer cells were collected and lysed using the VCX105 ultrasonic cell crusher (SONICS, Newtown, USA). Subsequently, the lactate concentrations in cell lysates were detected using the Lactate Assay Kit (MAK064, Sigma).

### Mitotracker-Red Staining

Briefly, breast cancer cells were treated with ursolic acid (10~20 μM) or 100 μM 3-BrPA for 24 h. Then, cells were stained with 100 nM Mitotracker-red solutions (M22425, Invitrogen) for 30 mins. After washing with PBS for 3 times, cells were observed under the fluorescent microscope. The fluorescence intensities of Mitotracker-red stained cells were measured using the NovoCyte flow cytometer.

### Oxygen Consumption Rates and Extracellular Acidification Rates Detection

Breast cancer cells were treated with 10 μM ursolic acid or 100 μM 3-BrPA for 24 h. The primary breast cancer cells were isolated from mice in the saline group and ursolic acid treatment group, respectively. The mitochondrial respiration function and glycolytic metabolism of the above cells were detected using the Seahorse XF24 extracellular flux analyzer (Seahorse Bioscience) as we described previously (18). The XF Cell Mito Stress Test Kit (103015-100, Agilent) was used to measure OCRs while the XF Glycolysis Stress Test Kit (103020-100, Agilent) was used to measure ECARs. Briefly, cells were seeded into XF24 cell culture microplates at a density of 4×10^4^ cells per well and cultured overnight. The XF24 cartridge was equilibrated with the calibration solution overnight at 37°C. The final concentrations of the cellular stress-inducing reagents used for OCRs detection were 10 mM for glucose, 2 mM for glutamine, 1 mM for sodium pyruvate, 1 μM for oligomycin, 1 μM for carbonyl cyanide 4-(trifluoromethoxy) phenylhydrazone (FCCP), 0.5 μM for antimycin A and 0.5 μM for rotenone, respectively. The final concentrations of the cellular stress-inducing reagents used for ECARs detection were 1 mM for glutamine, 10 mM for glucose, 1 μM for oligomycin, and 50 mM for 2-DG, respectively. All the reagents were loaded in the ports according to the manufacturer’s instructions.

### Transmission Electron Microscopy Observation

Cells were treated with 10 μM or 20 μM ursolic acid for 24 h. The caveolae structures in breast cancer cells were observed and digitally photographed by the transmission electron microscope as described previously (24).

### QPCR

QPCR assay was conducted as previously described (18). Briefly, breast cancer cells were treated with 1~30 μM ursolic acid for 48 h. Then, total RNA was extracted from breast cancer cells with Trizol and reverse transcribed to complementary cDNA using the PrimeScript™ RT reagent Kit (Takara, Shiga, Japan). PCR amplification reaction was performed using the SYBR Premix Ex Taq Kit (Takara) and the ABI 7500 Real-Time PCR System (Applied Biosystems, Foster City, CA, USA). Primer sequences of *CAV1* were 5′-GAGCGAGAAGCAAGTGTACGA-3′ (forward) and 5′- ACAGACGGTGTGGACGAAGAT-3′ (reverse). Primer sequences of *β-ACTIN* were 5′- CCAACCGCGAGAAGATGA-3′ (forward) and 5′- CCAGAGGCGTACAGGGATAG -3′ (reverse).

### Double Luciferase Reporter Gene Assay

Firstly, the *CAV1* promoter plasmid (HPRM37455, Genecopeia) was transfected into breast cancer cells using Vigenefection regnant (FH880806, Vigene Biosciences). Then, the transfected cells were seeded in the 96-well plate at a density of 1×10^4^ cells/well and were respectively treated with 10 μM ursolic acid, SP1 siRNA, or their combination for 24 h. Then, the cell culture supernatant of each well was collected. The *CAV1* promoter activity was detected by analyzing the gaussia luciferase activity and secreted alkaline phosphatase activity in the cell culture supernatant using the Secrete-Pair™ Dual Luminescence Assay Kit (LF031, Genecopeia) according to the manufacturer’s instructions (4).

### Chromatin Immunoprecipitation-PCR Assay

CHIP assay was conducted by immune-precipitating the DNA fragments with SP1 antibody (9389S, CST) using the CHIP Assay Kit (P2078, Beyotime) (25). Analysis of the genomic sequence of *CAV1* promoter (NC_000007.14:116523009-116525008) using the hTFtarget database revealed two potential binding sites (5’-GGGCGG-3’, site 1:-459 to -454 bp; site 2: -92 to -87 bp) for the transcription factor SP1. These two regions in the immune-precipitated DNA samples were amplified by PCR assay using the following primers including 5′-CCCTCTGTGAAACAGGGAGAC-3′ (forward), 5′- AAGGCGGCAGAAACAATCCA-3′ (reverse) and 5′-AACGTTCTCACTCGCTCTCTG-3′ (forward), 5′-TTTCCCTGGGCTGTGCTTT-3′ (reverse).

### Zebrafish Breast Cancer Xenotransplantation Model

The AB strain zebrafishes were obtained from China Zebrafish Resource Center. The zebrafish breast cancer xenotransplantation model was established as we previously described (15, 22). Briefly, MDA-MB-231 cells were collected in free culture medium and labeled red fluorescence by 5 μM 1,1′-Dioctadecyl-3,3,3′,3′-tetramethylindocarbocyanine perchlorate (DiI, Sigma-Aldrich). At 48 h post-fertilization, 200 Dil-stained MDA-MB-231 cells were suspended in 20 nl medium and injected into the perivitelline space of each embryo using a microinjector to establish the zebrafish breast cancer xenotransplantation model. The juvenile zebrafish bearing the Dil-stained breast cancer MDA-MB-231 cells were incubated in aquaculture water in 6-well plates and treated with 10 μM or 20 μM ursolic acid for 48 h. The effect of ursolic acid on breast cancer cell proliferation and metastasis in zebrafish was observed under the Nikon SMZ25 stereomicroscope.

### Animal Experiments

Animal studies were approved by the Institutional Animal Care and Use Committee of Guangdong Provincial Hospital of Chinese Medicine (No.2018065) and performed under our institutions’ instructions for the use of laboratory animals. Female Balb/c mice (18~22 g) were obtained from the Beijing Vital River Laboratory Animal Technology Co., Ltd. The 4T1-Luc breast cancer xenograft model was established by subcutaneously injecting 2×10^6^ 4T1-Luc cells into the mammary fat pads of mice (6). The tumor-bearing mice were randomly divided into 3 groups (n = 6), including saline group, 25 mg/kg/day ursolic acid group (intraperitoneally [i.p.]), and 50 mg/kg/day ursolic acid group. Mice were weighed and tumor volumes were measured every 3 days. Tumor volumes (V) were determined using the formula: V= (length × width^2^)/2. Mice were photographed using the IVIS Lumina XR *in vivo* imaging system (PerkinElmer) to monitor the growth and metastasis of 4T1-Luc xenografts. When tumors of the saline group reached a diameter of 2 cm, mice were euthanized by isoflurane. Then, lungs and tumors were excised and photographed. Primary breast cancer cells were isolated from fresh tumors using the mechanical method. Briefly, single-cell suspensions were prepared from tumors by slide mechanical grind. The red blood cells in samples were lysed and cells were re-suspended in the complete culture medium for further culture. Lung tissues and the remaining tumor tissues were preserved and subjected to tissue immunofluorescence assay or HE staining assay. To investigate the liver and kidney function toxicities as well as the hematologic toxicity of ursolic acid, the blood samples were collected and subjected for biochemical analysis as previously reported (22). Briefly, mouse serum (1:3 dilution) was obtained by centrifugation and used to analyze the hepatic function parameters (alanine transaminase and aspartate aminotransferase) and renal function parameters (urea, uric acid, and creatinine) using the Automatic Biochemical Analyzer (Roche Group, Basel, Switzerland). The fresh blood samples were collected and used to detect hematological parameters including white blood cell numbers, red blood cell numbers, and hemoglobin content using the Automatic Blood Cell Analyzer (Mindray, Shenzhen, China).

### HE Staining Assay

HE staining assay was conducted (22) to investigate the effect of ursolic acid on lung metastasis of breast cancer. Briefly, lung tissue sections were deparaffinized and hydrated. Then, 10% hematoxylin was used to visualize the cell nucleus, while 1% eosin was used to stain the cytoplasm. Finally, the specimens were dehydrated, cleared, and mounted for microscopic examination.

### Statistical Analysis

Statistics were calculated using the SPSS 26.0 software. Data were expressed as mean ± SD. ANOVA and Student’s t-test analyses were used for pairwise comparisons. *p*<0.05 was considered statistically significant.

## Results

### Bioactivity-Guided Fractionation Identifies Ursolic Acid as the Bioactive Compound of *Oldenlandia diffusa* Against Breast Cancer

**Figure 1:**
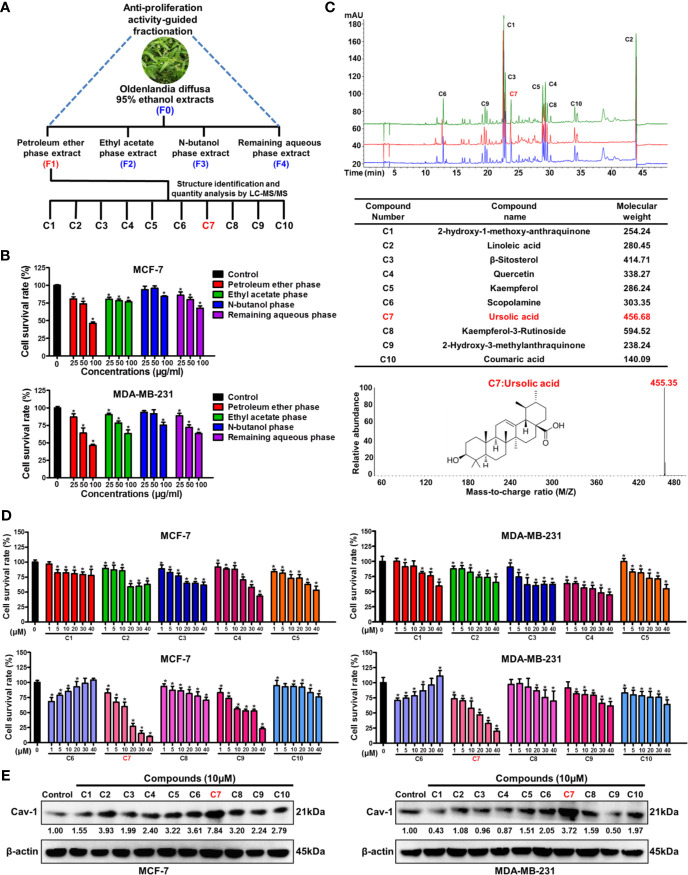
Bioactivity-guided fractionation identifies ursolic acid as the bioactive compound of *Oldenlandia diffusa* against breast cancer. **(A)** Schematic illustration of the bioactivity-guided fractionation procedure. **(B)** MCF-7 and MDA-MB-231 cells were treated with different extracts of *Oldenlandia diffusa* (25~100 μg/ml) for 48 h CCK8 assay was conducted to investigate the survival rates of breast cancer cells. **(C)** The top 10 most abundant compounds in the F1 fraction were identified and quantified by LC-MS/MS. **(D)** MCF-7 and MDA-MB-231 cells were treated with 10 kinds of compounds (1~40 μM) for 48 h CCK8 assay was conducted to investigate the survival rates of breast cancer cells. **(E)** The expression change of Cav-1 protein in MCF-7 and MDA-MB-231 cells after treatment with 10 kinds of compounds (10 μM) for 48 h N = 3. ^*^ *p* < 0.05.

Bioactivity-guided fractionation is the commonly used method to separate the bioactive constituents of herbs or TCM formulas (6). To determine the material basis accounting for the anticancer activity of *Oldenlandia diffusa*, bioactivity-guided fractionation was firstly applied based on the inhibitory effect on breast cancer cell proliferation. Firstly, the ethanol extracts (F0) of *Oldenlandia diffusa* were isolated by the refluxing extraction method and partitioned into four fractions depending on the solvent polarity differences (**Figure 1A**). The first round of bioactivity screening identified the F1 fraction (petroleum ether phase extracts) as the target subset since it exhibited the strongest anti-proliferation activity in both MCF-7 and MDA-MB-231 cells (**Figure 1B**). Therefore, the compound composition of the F1 fraction was further analyzed by LC-MS/MS detection (**Figure 1C**). By comparing the peak areas, the top 10 most abundant compounds in the F1 fraction were identified as 2-hydroxy-3-methylanthraquinone (C1), linoleic acid (C2), β-sitosterol (C3), quercetin (C4), kaempferol (C5), scopolamine (C6), ursolic acid (C7), kaempferol-3-rutinoside (C8), 2-hydroxy-3-methylanthraquinone (C9) and coumaric acid (C10). Among these 10 compounds, ursolic acid (1~40 μM) exhibited the strongest anti-proliferative activity in both MCF-7 and MDA-MB-231 cells (**Figure 1D**). Our previous studies have identified Cav-1 as a promising cancer suppressor protein as it could impair breast cancer growth and metastasis by impairing glycolytic metabolism (18, 22). Notably, among the 10 compounds, ursolic acid also exhibited the strongest induction effect on Cav-1 expression in breast cancer cells (**Figure 1E** and **Supplementary Figure 1**). Altogether, the above results suggest ursolic acid as the potential bioactive compound of *Oldenlandia diffusa* against breast cancer.

### Ursolic Acid Significantly Restrains the Proliferation and Induces Mitochondrial-Mediated Apoptosis of Breast Cancer Cells

**Figure 2:**
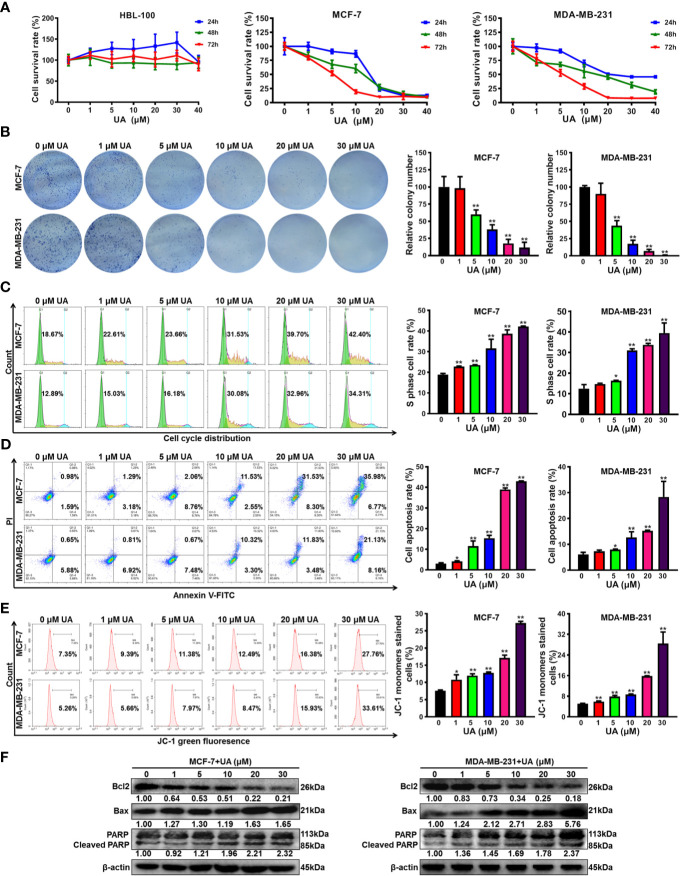
Ursolic acid significantly restrains the proliferation and induces mitochondrial-mediated apoptosis of breast cancer cells *in vitro*. **(A)** HBL-100, MCF-7 and MDA-MB-231 cells were treated with 1~40 μM ursolic acid for 24~72 h The cytotoxicity of ursolic acid in different cell lines was detected by CCK8 assay. **(B)** Colony formation assay was conducted to investigate the effect of ursolic acid (1~30 μM) on the clonogenic activity of breast cancer cells following a 10-day culture period. **(C)** The cell cycle distributions of MCF-7 and MDA-MB-231 cells after ursolic acid (1~30 μM) treatment for 48 h were detected by flow cytometry. **(D)** The apoptosis ratios of MCF-7 and MDA-MB-231 cells after ursolic acid (1~30 μM) treatment for 48 h were detected by Annexin V-FITC/PI staining and flow cytometry. **(E)** The mitochondrial depolarization and apoptosis of MCF-7 and MDA-MB-231 cells after ursolic acid (1~30 μM) treatment for 48 h were detected by JC-1 staining assay and flow cytometry. **(F)** The expression changes of the indicated proteins in MCF-7 and MDA-MB-231 cells after ursolic acid (1~30 μM) treatment for 48 h N = 3. ^*^ *p* < 0.05. ^**^ *p* < 0.01.

To convince the anticancer activity of ursolic acid, the anti-proliferative and pro-apoptotic effects of ursolic acid on breast cancer cells were further investigated *in vitro*. Ursolic acid (1~40 μM) significantly suppressed the proliferation of breast cancer cells in a concentration-time-dependent manner. The IC_50_ values of ursolic acid were 7.96 μM for MCF-7 cells and 9.02 μM for MDA-MB-231 cells at 48 h, respectively. In contrast, ursolic acid exhibited little cytotoxicity in the nonmalignant mammary epithelial HBL-100 cells (**Figure 2A** and **Supplementary Figure 2**). Similarly, ursolic acid (1~30 μM) also dramatically restrained the clonogenic activity of breast cancer cells following a 10-day culture period (**Figure 2B**). Furthermore, cell cycle distribution assay indicated that ursolic acid (1~30 μM) could significantly induce S-phase arrest of breast cancer cells (**Figure 2C**). Moreover, ursolic acid (1~30 μM) could strongly induce the apoptosis of breast cancer cells (**Figure 2D**). JC-1 staining assay further suggested that ursolic acid (1~30 μM) could induce mitochondrial depolarization and apoptosis of breast cancer cells (**Figure 2E**), which was achieved by attenuating the anti-apoptotic protein Bcl2 whereas elevating the pro-apoptotic proteins Bax and cleaved PARP (**Figure 2F** and **Supplementary Figure 3**). Altogether, ursolic acid significantly restrains the proliferation and induces mitochondrial-mediated apoptosis of breast cancer cells *in vitro*.

### Ursolic Acid Impairs the Glycolytic Metabolism and Mitochondrial Respiration Function of Breast Cancer Cells

**Figure 3:**
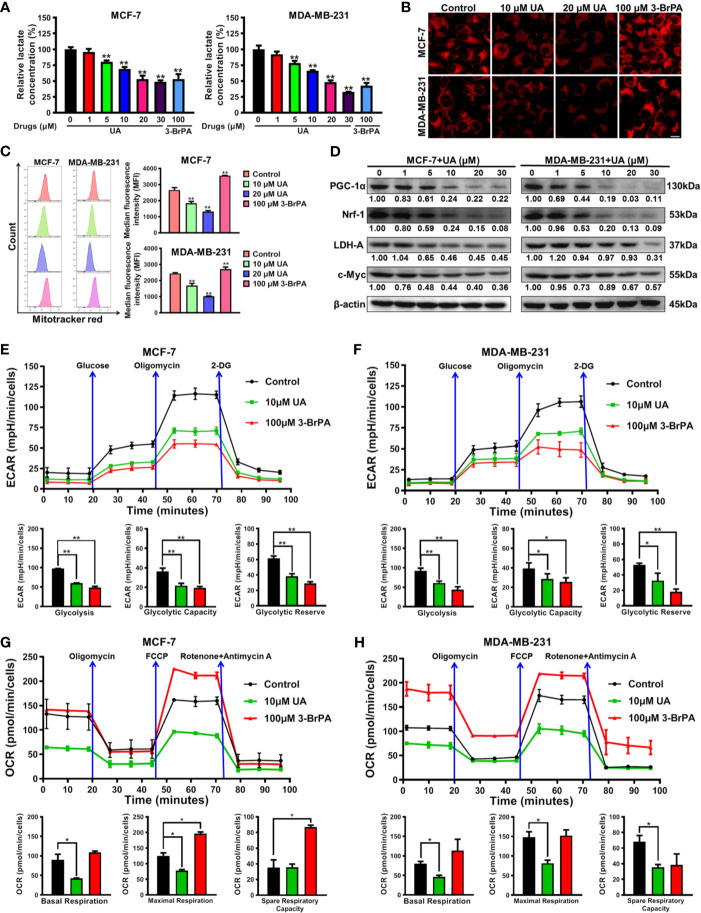
Ursolic acid impairs the glycolytic metabolism and mitochondrial respiration function of breast cancer cells. **(A)** MCF-7 and MDA-MB-231 cells were treated with 1~30 μM ursolic acid or 100 μM 3-BrPA for 48 h The lactate concentrations in cell lysates were detected using the Lactate Assay Kit. **(B, C)** Breast cancer cells were treated with 10~20 μM ursolic acid or 100 μM 3-BrPA for 24 h Mitotracker-red staining assay and flow cytometry were conducted to investigate the accumulation changes of Mitotracker-red in mitochondria of breast cancer cells. Scale bar = 10 μm. **(D)** The expression changes of the indicated proteins in MCF-7 and MDA-MB-231 cells after ursolic acid (1~30 μM) treatment for 48 h **(E–H)** MCF-7 and MDA-MB-231 cells were treated with 10 μM ursolic acid or 100 μM 3-BrPA for 24 h The Seahorse XF24 extracellular flux analyzer was used to detect OCRs to measure mitochondrial respiration, and to detect ECARs to determine glycolysis. N = 3. ^*^ *p* < 0.05. ^**^ *p* < 0.01.

Next, the biological mechanism underlying the inhibitory activity of ursolic acid in breast cancer cells was investigated. Previous studies have proven that Cav-1 is closely implicated in the metabolic modulation of breast cancer cells (21) and could impair their glycolytic activity (18, 22). As stated above, ursolic acid could significantly elevate Cav-1 expression and induce mitochondrial depolarization in breast cancer cells. These findings implicated that ursolic acid may inhibit breast cancer by impairing the glycolytic metabolism and mitochondrial respiration function of breast cancer cells. Lactate is the metabolic ending product of glycolytic metabolism (18). Similar to the effect of 3-BrPA (glycolytic inhibitor), ursolic acid (5~30 μM) treatment also dramatically decreased lactate production from breast cancer cells (**Figure 3A**), implying that ursolic acid suppressed the glycolytic metabolism of breast cancer cells. Furthermore, the effect of ursolic acid on the mitochondrial respiration function of breast cancer cells was determined by staining functional mitochondria with Mitotracker-red, whose accumulation depends on mitochondrial membrane potential (18, 26). Ursolic acid (10~20 μM) treatment also significantly decreased Mitotracker-red accumulation in mitochondria of breast cancer cells (**Figures 3B, C**), which was consistent with the above results that ursolic acid could induce mitochondrial depolarization and apoptosis of breast cancer cells. These results suggested that ursolic acid also impaired the mitochondrial respiration function of breast cancer cells. Western blotting assay (**Figure 3D** and **Supplementary Figure 4**) further convinced the above findings as ursolic acid (1~30 μM) treatment significantly attenuated the protein expression levels of both the glycolysis-related proteins (c-Myc and LDH-A) and the mitochondrial respiration-related proteins (Nrf1 and PGC-1α). Cell energy metabolism analyzer is the current “gold standard” method for detecting the metabolic activity changes of cells, which detects OCRs to measure mitochondrial respiration and ECARs to measure glycolysis. Similar to the effect of 3-BrPA, ursolic acid treatment impaired the glycolytic metabolism in terms of glycolysis, glycolytic capacity, and glycolytic reserve in breast cancer cells (**Figures 3E, F**). Meanwhile, ursolic acid treatment also impaired mitochondrial respiration in terms of basal respiration and maximal respiration in breast cancer cells (**Figures 3G, H**). Altogether, ursolic acid impairs both the glycolytic metabolism and mitochondrial respiration function of breast cancer cells *in vitro*.

### Ursolic Acid Impairs the Glycolytic Metabolism of Breast Cancer Cells by Activating Cav-1 Signaling

**Figure 4:**
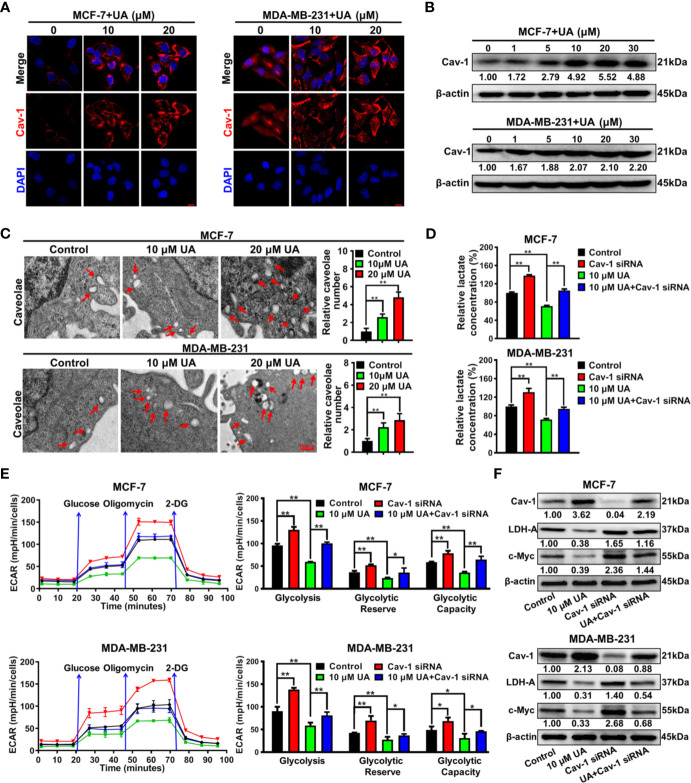
Ursolic acid impairs the glycolytic metabolism of breast cancer cells by activating Cav-1 signaling. **(A)** MCF-7 and MDA-MB-231 cells were treated with 10~20 μM ursolic acid for 48 h Cell immunofluorescence assay was conducted to investigate the effect of ursolic acid on Cav-1 expression in breast cancer cells. Scale bar=10 μm. **(B)** The expression change of Cav-1 protein in MCF-7 and MDA-MB-231 cells after ursolic acid (1~30 μM) treatment for 48 h **(C)** MCF-7 and MDA-MB-231 cells were treated with 10~20 μM ursolic acid for 24 h The caveolae structures in breast cancer cells were observed by the transmission electron microscope. **(D–F)** Breast cancer cells were transfected with Cav-1 specific siRNAs or treated with 10 μM ursolic acid for 48 h The combinational effects of Cav-1 knockdown and ursolic acid treatment on lactate production **(D)**, ECARs **(E)** and glycolysis-related proteins **(F)** of breast cancer cells were detected. N = 3. ^*^ *p* < 0.05. ^**^ *p* < 0.01.

Our previous studies have proven that Cav-1 could suppress the glycolytic metabolism of breast cancer cells, and therefore represents a reliable target for glycolysis-related treatment strategies (18, 22). It was found that ursolic acid treatment strongly elevated Cav-1 expression in both MCF-7 and MDA-MB-231 cells (**Figures 4A, B** and **Supplementary Figure 5**). Cav-1 is a structural protein of 50~100 nm membrane invaginations called caveolae. *In situ* observations indicated that ursolic acid treatment increased the numbers of caveolae in breast cancer cells (**Figure 4C**). These results proved the up-regulation effect of ursolic acid on Cav-1 expression in breast cancer cells. To convince that Cav-1 is the molecular target of ursolic acid in impairing the glycolytic metabolism, the combination effect of Cav-1 siRNAs and ursolic acid on glycolytic metabolism of breast cancer cells was explored. It was found that Cav-1 knockdown by Cav-1 specific siRNAs could significantly promote lactate production, ECARs and the expression levels of glycolysis-associated proteins in breast cancer cells. More importantly, Cav-1 knockdown partly abrogated the inhibitory effects of ursolic acid on that in breast cancer cells (**Figures 4D-F** and **Supplementary Figure 6**). In terms of cell growth and death, Cav-1 knockdown also partly abrogated both the growth inhibitory effect and the apoptosis induction effect of ursolic acid in breast cancer cells (**Supplementary Figures 7, 8** **).** Altogether, ursolic acid impairs the glycolytic activity of breast cancer cells by activating Cav-1 signaling.

### Ursolic Acid Impairs the Glycolytic Metabolism of Breast Cancer Cells by Activating SP1/Cav-1 Signaling

**Figure 5:**
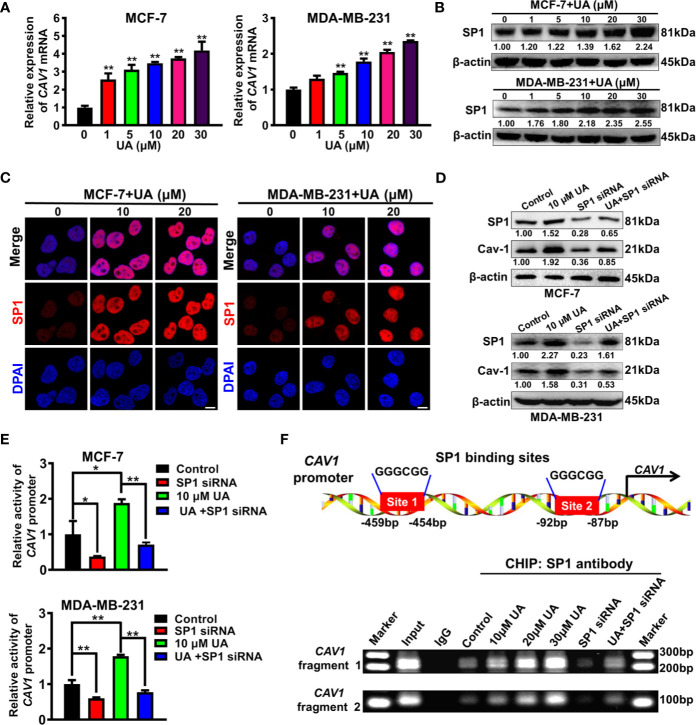
Ursolic acid impairs the glycolytic metabolism of breast cancer cells by activating SP1/Cav-1 signaling. **(A)** MCF-7 and MDA-MB-231 cells were treated with 1~30 μM ursolic acid for 48 h QPCR assay was conducted to investigate the effect of ursolic acid on *CAV1* mRNA expression in breast cancer cells. **(B)** The expression change of SP1 protein in MCF-7 and MDA-MB-231 cells after ursolic acid (1~30 μM) treatment for 48 h **(C)** MCF-7 and MDA-MB-231 cells were treated with 10~20 μM ursolic acid for 48 h Cell immunofluorescence assay was conducted to investigate the effect of ursolic acid on SP1 expression in breast cancer cells. Scale bar = 10 μm. **(D)** Breast cancer cells were transfected with SP1 specific siRNAs or treated with 10 μM ursolic acid for 48 h The combinational effects of SP1 knockdown and ursolic acid treatment on SP1 and Cav-1 expression levels in breast cancer cells were detected by Western blotting assay. **(E)** Breast cancer cells were treated with 10 μM ursolic acid for 24 h or transfected with SP1 specific siRNAs. The combinational effects of SP1 knockdown and ursolic acid treatment on *CAV1* promoter activity in breast cancer cells were detected by the double luciferase reporter gene assay. **(F)** MCF-7 cells were transfected with SP1 specific siRNAs or treated with 10~30 μM ursolic acid for 48 h CHIP-PCR assay was conducted to investigate the effects of ursolic acid treatment, SP1 knockdown or their combination on the binding of SP1 with *CAV1* promoter region of breast cancer cells. N = 3. ^*^ *p* < 0.05. ^**^ *p* < 0.01.

Next, the molecular mechanism underlying the induction effect of ursolic acid on Cav-1 expression in breast cancer cells was investigated. It was found that ursolic acid treatment could significantly increase *CAV1* mRNA levels in breast cancer cells in a concentration-dependent manner (**Figure 5A**), suggesting that ursolic acid could induce Cav-1 expression by elevating its transcription activity. To determine which transcription factor participated in this process, we proceeded to predict the potential transcription factors of *CAV1* gene using the hTFtarget database. Analysis of the genomic sequence of *CAV1* promoter (NC_000007.14:116523009-116525008) revealed two potential binding sites (5’-GGGCGG-3’, site 1:-459 to -454 bp; site 2: -92 to -87 bp) for the transcription factor SP1. Therefore, ursolic acid may activate the transcription activity of *CAV1* gene by activating SP1 signaling. To validate this conjecture, the modulatory effect of ursolic acid on SP1 expression was further detected. Ursolic acid treatment could significantly promote SP1 expression in breast cancer cells (**Figures 5B, C** and **Supplementary Figure 9**). What is more, SP1 knockdown could partially abrogate the induction effect of ursolic acid on both Cav-1 protein expression (**Figure 5D** and **Supplementary Figure 10**) and *CAV1* promoter activity (**Figure 5E**) in breast cancer cells. More importantly, CHIP-PCR assay validated that ursolic acid could promote the binding of SP1 with *CAV1* promoter region of breast cancer cells, while SP1 knockdown could partially abrogate that (**Figure 5F**). These results indicated that ursolic acid could transcriptionally activate *CAV1* signaling by inducing the expression of transcription factor SP1. Altogether, ursolic acid impairs the glycolytic metabolism of breast cancer cells by activating SP1/Cav-1 signaling.

### Ursolic Acid Inhibits Breast Cancer Growth and Metastasis in Both the Zebrafish and Mouse Breast Cancer Xenotransplantation Models Without Observable Toxicities

**Figure 6:**
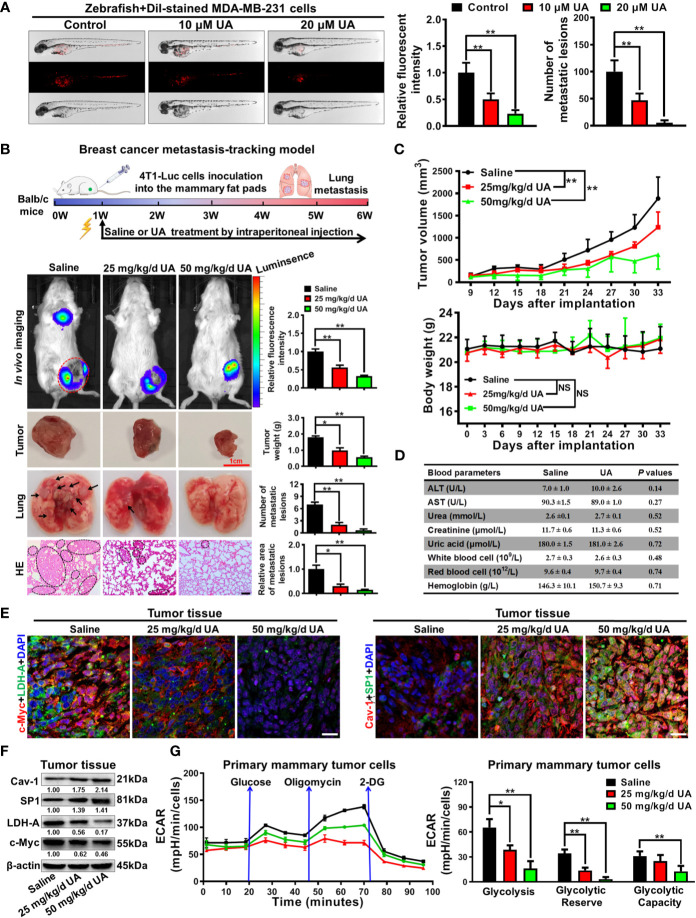
Ursolic acid inhibits breast cancer growth and metastasis in zebrafish and mouse xenotransplantation models of breast cancer without observable toxicities. **(A)** The juvenile zebrafish bearing the Dil-stained breast cancer MDA-MB-231 cells were treated with 10~20 μM ursolic acid for 48 h The proliferation and dissemination of the injected MDA-MB-231 cells in the zebrafish breast cancer xenotransplantation model were observed under a stereomicroscope. N = 3. **(B, C)** The 4T1-Luc breast cancer xenograft model was established by subcutaneously injecting 4T1-Luc cells into the mammary fat pads of mice. Ursolic acid administration (25~50 mg/kg/d) by intraperitoneal injection suppressed the growth and lung metastasis of 4T1-Luc xenografts in mouse breast cancer xenotransplantation models. N = 6. Scale bar = 100 μm. **(D)** The hepatotoxicity, nephrotoxicity and hematotoxicity of ursolic acid in mice were detected by biochemical analysis. N = 3. **(E, F)** Tissue immunofluorescence assay **(E)** and Western blotting assay **(F)** were conducted to investigate the effect of ursolic acid on the expression levels of LDH-A, c-Myc, SP1 and Cav-1 in mammary tumor tissues. N = 3. Scale bar = 20 μm. **(G)** The effect of ursolic acid on glycolytic metabolism of primary breast cancer cells was investigated by measuring ECARs. N = 3. ^*^ *p* < 0.05. ^**^ *p* < 0.01.

**Figure 7:**
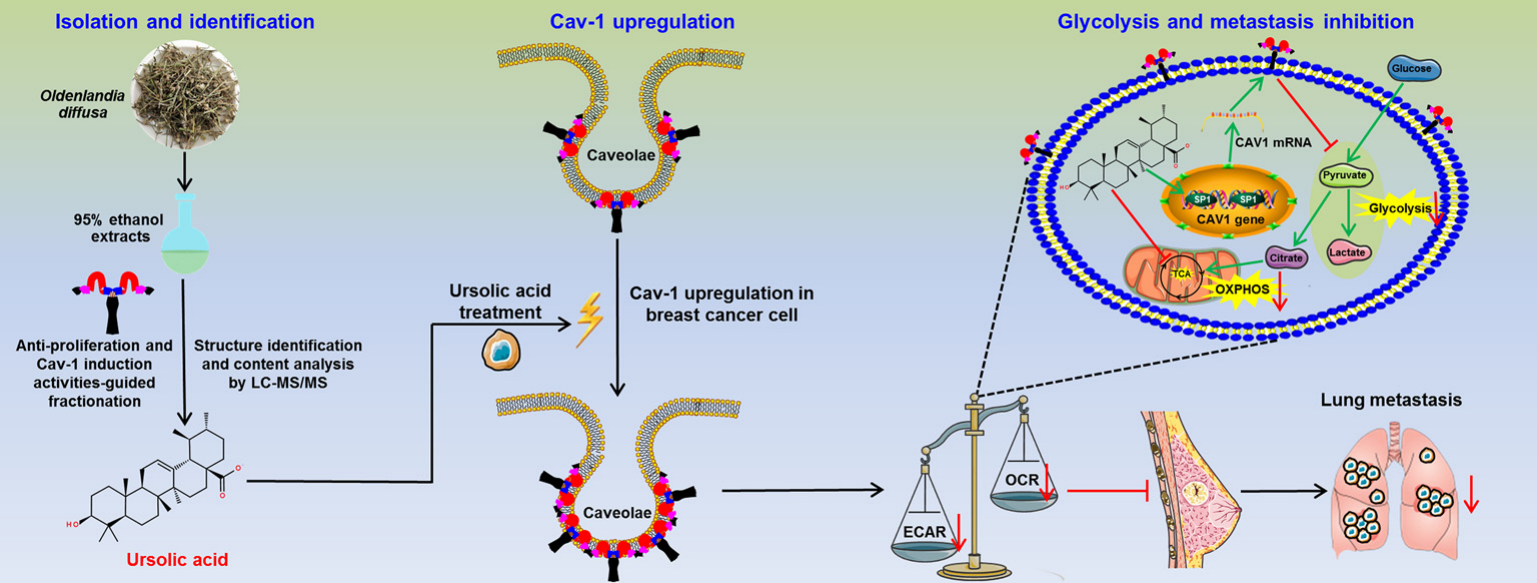
The schematic diagram of this study. Ursolic acid is identified as the bioactive compound of *Oldenlandia diffusa* against breast cancer. Ursolic acid inhibits breast cancer metastasis by suppressing glycolytic metabolism *via* activating SP1/Caveolin-1 signaling.

Lastly, the anti-breast cancer activities of ursolic acid and the underlying mechanisms were validated *in vivo*. It was found that ursolic acid (10~20 μM) treatment dramatically decreased the proliferation and dissemination of the injected MDA-MB-231 cells in the zebrafish xenotransplantation model, suggesting that ursolic acid could also restrain breast cancer growth and metastasis *in vivo* (**Figure 6A**). To further confirm this finding, an orthotopic mammary tumor xenograft model was constructed by injecting 4T1-Luc cells into the fat pads of mice *in situ*. Ursolic acid administration (25~50 mg/kg/d) by intraperitoneal injection could also remarkably suppress the growth and lung metastasis of 4T1-Luc xenografts in the mouse xenotransplantation model of breast cancer. Meanwhile, no treatment-associated mortality or significant decline in body weight was observed (**Figures 6B, C**). To further evaluate the biosafety of ursolic acid, blood samples of mice were collected and subjected to blood biochemical and blood routine analyses. It was found that ursolic acid treatment had no observable hepatotoxicity, nephrotoxicity or hematotoxicity in mice (**Figure 6D**). These results highlighted ursolic acid as a very efficient and low-toxic candidate compound for breast cancer treatment. In terms of mechanisms, both immunofluorescence assay and western blotting assay validated that ursolic acid treatment significantly attenuated the expression levels of glycolysis-associated proteins (LDH-A and c-Myc) whereas increasing SP1 and Cav-1 expression levels in mammary tumor tissues (**Figures 6E, F** and **Supplementary Figure 11**). More importantly, ursolic acid administration also significantly suppressed the glycolytic metabolism under conditions of glycolysis, glycolytic capacity and glycolytic reserve in primary mammary tumor cells *in vivo* (**Figure 6G**). Taken together, ursolic acid hinders breast cancer growth and metastasis *in vivo* by suppressing glycolytic metabolism *via* activating SP1/Cav-1 signaling (**Figure 7**).

## Discussion

Despite the significant improvements in treatment strategy and survival prolongation (2), breast cancer still represents the most serious malignancy among women in terms of both incidence and mortality worldwide (3). 90% of breast cancer-related deaths result from distant metastases in lung, bone, liver and brain while lung metastasis alone leads to 60~70% of breast cancer-related deaths (27, 28). At present, metastatic breast cancer is almost incurable of which the median 5-year survival is only about 26% (29). Therefore, novel agents for breast cancer prevention and treatment, particularly breast cancer metastasis, are urgently needed. Nowadays, medical herbs have been particularly acknowledged as a crucial source for the discovery and development of novel anticancer drugs. With proven efficacy and safety, *Oldenlandia diffusa* has been used as one of the most frequently prescribed medical herbs for breast cancer treatment in TCM (6, 10–14). Herein, bioactivity-guided fractionation identified ursolic acid as the bioactive compound of *Oldenlandia diffusa* in suppressing breast cancer. Further investigations revealed that ursolic acid suppressed breast cancer growth and lung metastasis by impairing glycolytic metabolism *via* inducing the SP1/Cav-1 pathway. The present study not only provides the experimental basis for the anticancer application of *Oldenlandia diffusa* in the clinic but also presents ursolic acid as a potential glycolytic inhibitor for breast cancer glycolytic modulation. Additionally, the fingerprint analysis results indicated that ursolic acid was one of the most abundant phytochemicals in *Oldenlandia diffusa*. Previous studies also reported that the average content of ursolic acid in the fresh *Oldenlandia diffusa* was as high as 7.293 ± 2.776 mg/g (30). More importantly, ursolic acid has been reported to exhibit a wide antitumor spectrum and could inhibit multiple solid tumors including breast cancer (31, 32), colorectal cancer (33, 34), cervical cancer (35), pancreatic cancer (36) and prostate cancer (37). Several clinical trials have also suggested that ursolic acid supplementation was well-tolerated with manageable toxicity, and could potentially improve patient remission rates in subjects with advanced solid tumors (38, 39). Altogether, the above findings strongly highlight ursolic acid as an easily available and effective therapy for cancer treatment. It should be noted that the existing clinical trials investigating the antitumor activity of ursolic acid are still relatively limited. Therefore, more clinical trials are highly recommended to further verify the biosafety and antitumor activity of ursolic acid.

Glycolytic inhibitors represent a promising therapeutic strategy against cancer (40). However, the existing glycolytic inhibitors were greatly limited by their off-target effects and systemic toxicities. For example, 3-BrPA administration could lead to considerable toxicities in both the liver and gastrointestinal system *in vivo*, and even deaths at high doses (41, 42). Therefore, discovering low-cytotoxic glycolytic inhibitors is of an urgent need for cancer prevention and therapy and has attracted increasing research attentions. Ursolic acid is a natural pentacyclic triterpenoid with abundant content in various medical herbs (e.g. *Oldenlandia diffusa* and *Rosmarinus officinalis*). It has been reported that ursolic acid possessed diverse biological and pharmacological properties including anticancer, anti-microbial, anti-inflammatory, anti-angiogenic and antioxidant activities (43). In terms of antitumor mechanisms, accumulating studies have reported that ursolic acid could inhibit cancer proliferation, invasion, angiogenesis, metastasis (34) as well as inducing cell cycle arrest, apoptosis (44) and autophagy-dependent death of cancer cells (35). The molecular targets and signaling pathways involved in the anti-tumor activity of ursolic acid include Bcl2/Bax, Caspases, PARP, MAPKs, mTOR, AKT, COX2, p53, p21, NF-κB, JAK2/STAT3, VEGF, HIF-1α and so on (45). Furthermore, ursolic acid could also suppress cancer growth and metastasis by modulating the tumor microenvironment *via* reducing the infiltrations of myeloid-derived suppressor cells (MDSCs) and regulatory T cells (Tregs) (46). Moreover, ursolic acid could synergistically chemosensitize tumors to cytotoxic drugs including oxaliplatin (33) and capecitabine (34). To our best knowledge, the novel biological function of ursolic acid as a glycolytic inhibitor has been rarely reported. Herein, we demonstrated that ursolic acid could dramatically suppress the glycolytic metabolism of breast cancer both *in vitro* and *in vivo*. More importantly, ursolic acid not only exhibited low cytotoxicity in nonmalignant breast epithelial cells *in vitro* but also brought no detectable hepatotoxicity, nephrotoxicity or hematotoxicity *in vivo*. These results suggest that ursolic acid may be an ideal glycolytic inhibitor with low toxicity and economical characteristic. Noteworthy, it has been reported that ursolic acid is highly hydrophobic which leads to its disappointing systemic bioavailability and relatively short half-life in the circulation (31, 46). To improve its clinical applicability, ursolic acid-loaded formulations such as liposomes, nanoliposomes, nanocrystals, solid dispersions and nanoparticles are strongly encouraged. Notably, ursolic acid-loaded nanoparticles (31) and liposomes (46) have been successfully prepared recently, and were found to be very effective in solubilizing and delivering ursolic acid. It has been reported that the peak plasma concentration was 4153.4 ng/ml (approximately 9.1 μM) in humans given a single injection of ursolic acid nanoliposomes at an average dose of 98 mg/m^2^ body surface area (an average is 1.7 m^2^ for adult males) (47). Notably, ursolic acid is usually administrated at repetitive doses of 150 mg/day in clinical trials. In the present study, ursolic acid at concentrations above 5 μM significantly inhibited the glycolytic metabolism and growth of breast cancer cells *in vitro*. Therefore, the effective concentrations of ursolic acid as a glycolytic inhibitor may be pharmacologically achievable *in vivo*.

Consistent with our finding, multiple independent researchers have suggested that Cav-1 usually exhibited decreased expression in breast cancer cells than normal cells (18) while Cav-1 loss usually predicated a poor clinical outcome of breast cancer patients (48–50). Additionally, increasing studies also revealed that Cav-1 downregulation or loss usually occurred in tumor tissues of multiple transgenic breast cancer-prone mice (51). *CAV1* loss in MMTV-PyVT mice could accelerate the tumorigenesis and increase the tumor multiplicity and burden of breast cancer (18, 52, 53). Cav-1 is also closely implicated in the glycolytic metabolism of cancer cells. It has been reported that Cav-1 served as a binding protein for various rate-limiting enzymes of glycolytic metabolism including phosphofructokinase and aldolase (54). Additionally, Cav-1 could significantly inhibit the glycolytic metabolism of cancer cells by inducing the degradation of c-Myc (18). Meanwhile, pharmacological induction of Cav-1 signaling in cancer cells or cancer-associated fibroblasts by small molecule drugs (e.g. betulinic acid, quercetin, N-acetyl-cysteine and metformin) could significantly inhibit their glycolytic activities and therefore suppress cancer growth and metastasis (21, 22, 55). These results suggested that Cav-1 may represent an excellent screening target for glycolytic inhibitor identification. In the present study, we also delineated the molecular mechanism of ursolic acid-induced Cav-1 up-regulation in breast cancer cells. Previous studies have reported that transcription factor SP1 was essential for the transactivation of the *CAV1* gene (56, 57). However, the specific molecular mechanisms remain unclear. Herein, genomic sequence analysis further indicated that the *CAV1* promoter contained two GC-boxes (GGGCGG) for SP1 binding. Ursolic acid could transcriptionally elevate Cav-1 expression by promoting SP1 expression and its binding with these two sites in the *CAV1* promoter region. This finding provides the novel role of SP1 in controlling CAV1 expression and glycolysis inhibition.

## Conclusion

Taken together, ursolic acid, the bioactive compound of *Oldenlandia diffusa*, could dramatically suppress breast cancer growth and metastasis by impairing glycolytic metabolism *via* activating SP1/Cav-1 signaling. This study not only highlights ursolic acid as a promising candidate drug for breast cancer treatment but also sheds novel light on Cav-1 as a druggable target for glycolytic modulation of breast cancer.

## Data Availability Statement

The original contributions presented in the study are included in the article/**Supplementary Material**. Further inquiries can be directed to the corresponding authors.

## Ethics Statement

The animal study was reviewed and approved by the Institutional Animal Care and Use Committee of Guangdong Provincial Hospital of Chinese Medicine (No.2018065).

## Author Contributions

ZW and HO conceived and designed the experiments. SW and XC performed the experiments and wrote the manuscript. JZ, JL, NW, BY, BP, YZ and XW took part in the discussion and proofreading the manuscript. All authors contributed to the article and approved the submitted version.

## Funding

This work was supported by the National Natural Science Foundation of China [82174165, 82074165, 81873306, 81973526, 82004132, 82004373, 82060655 and 81703749]; the State Key Laboratory of Dampness Syndrome of Chinese Medicine [SZ2021ZZ19]; Guangdong Science and Technology Department [2021A0505030059, 2017B030314166, 2016A030306025]; Medical and Health program of Panyu Science & Technology Plan [2019-Z04-05]; Department of Education of Guangdong Province [2018KZDXM022, A1-2606-19-111-009, 2019KQNCX019]; The 2020 Guangdong Provincial Science and Technology Innovation Strategy Special Fund (Guangdong-Hong Kong-Macau Joint Lab) [2020B1212030006]; Guangdong Traditional Chinese Medicine Bureau Project [20201132, 20211114]; Guangzhou Science and Technology Project [202102010316, 202102010241, 201904010407]; The Specific Research Fund for TCM Science and Technology of Guangdong provincial Hospital of Chinese Medicine [YN2018MJ07, YN2018QJ08], and the Foundation for Young Scholars of Guangzhou University of Chinese Medicine [QNYC20190101].

## Conflict of Interest

The authors declare that the research was conducted in the absence of any commercial or financial relationships that could be construed as a potential conflict of interest.

## Publisher’s Note

All claims expressed in this article are solely those of the authors and do not necessarily represent those of their affiliated organizations, or those of the publisher, the editors and the reviewers. Any product that may be evaluated in this article, or claim that may be made by its manufacturer, is not guaranteed or endorsed by the publisher.
