# Supplementary material for: Ursolic Acid Inhibits Breast Cancer Metastasis by Suppressing Glycolytic Metabolism via Activating SP1/Caveolin-1 Signaling
Source: Front Oncol. 2021 Sep 8;11:745584. doi: 10.3389/fonc.2021.745584 (PMC8457520; doi:10.3389/fonc.2021.745584)
Supplement: Supplementary file 1 [file DataSheet_1.pdf]

Supplementary Material

1 Supplementary Figures

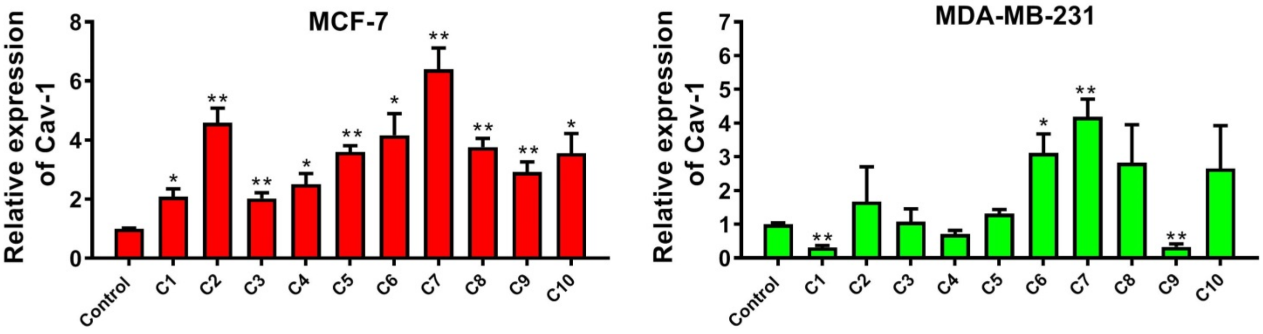

Supplementary Figure 1. Statistical analyses of the relative protein expression levels of the bands in Figure 1E. Relative protein expression levels were quantified by densitometric analyses of protein bands using the Gel-Pro analyzer 4 software. N = 3, $^{*}P < 0.05$, $^{**}P < 0.01$.

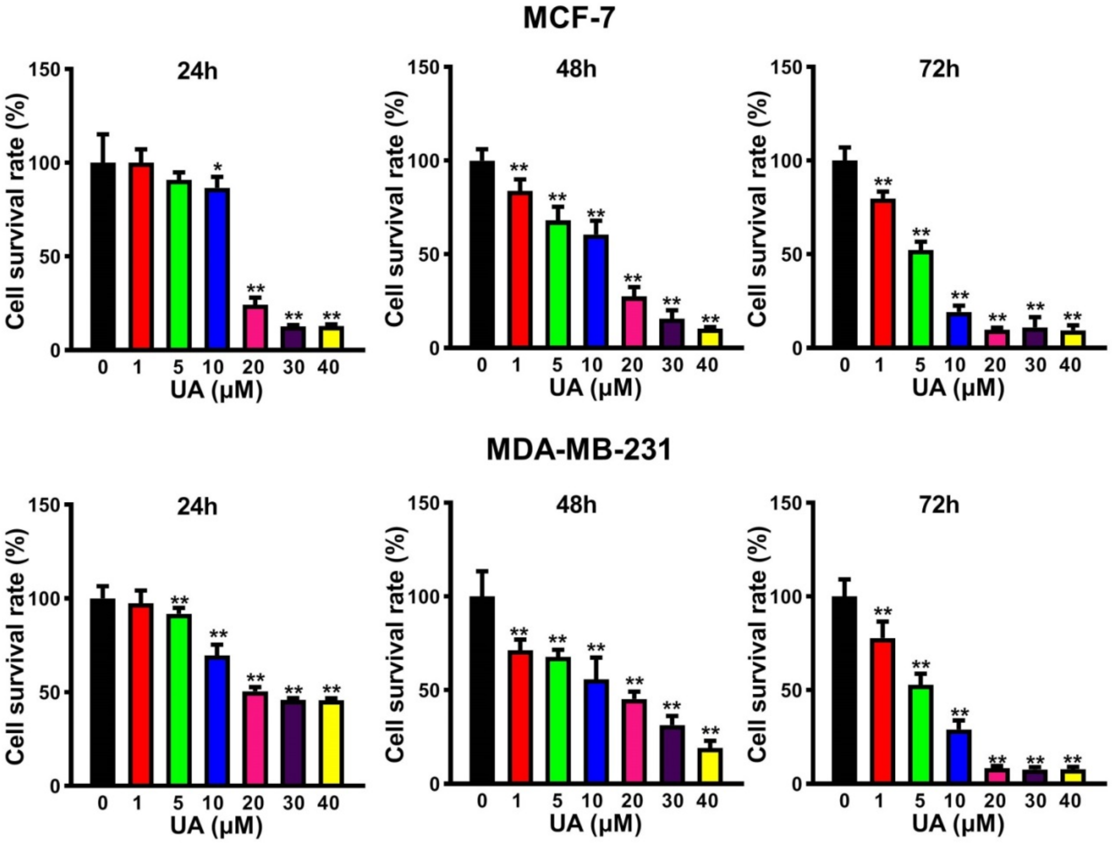

Supplementary Figure 2. The statistical analyses of cell survival rates of breast cancer cells. MCF-7 and MDA-MB-231 cells were treated with 1~40 μM ursolic acid for 24~72 h. CCK8 assay was conducted to detect their survival rates. N = 8, $^{*}P < 0.05$, $^{**}P < 0.01$.

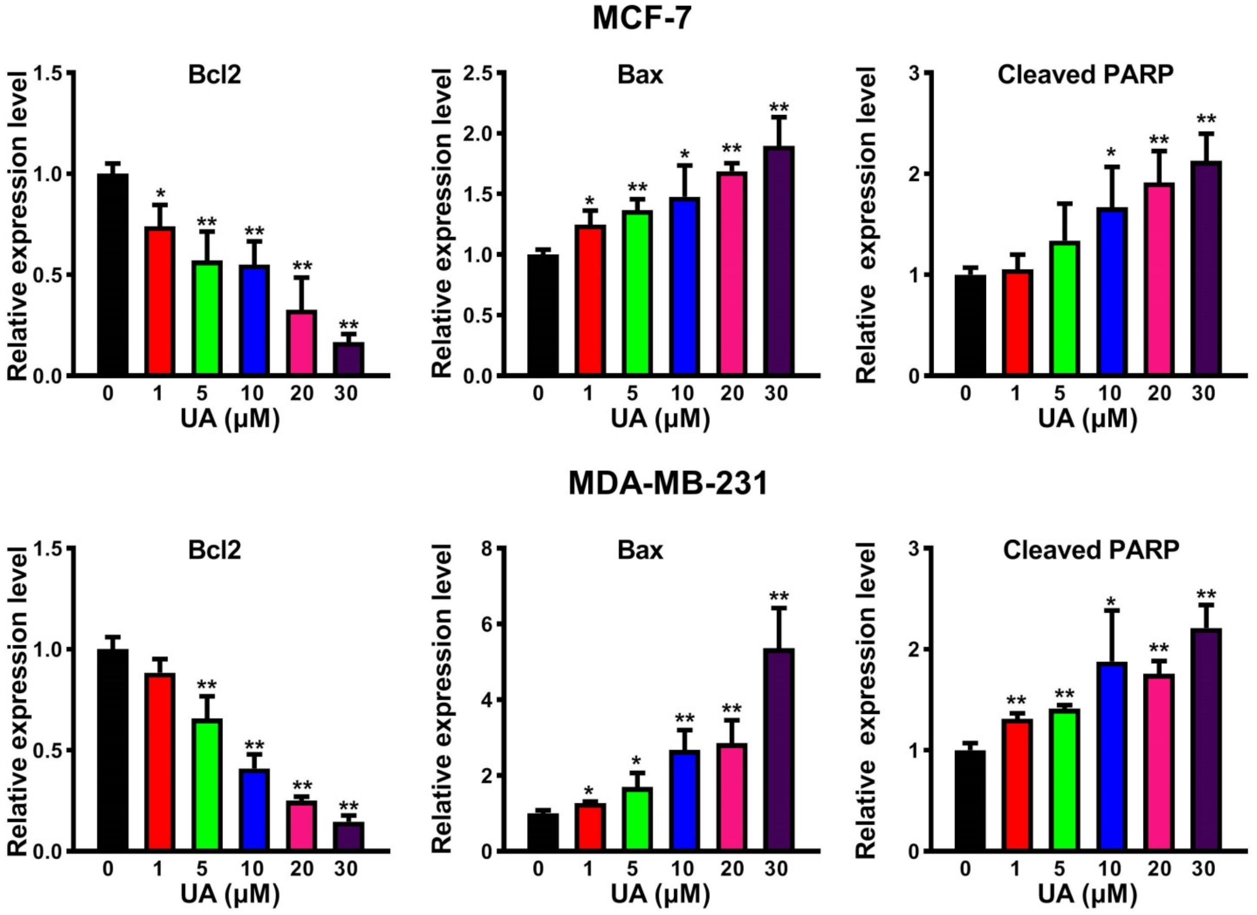

Supplementary Figure 3. Statistical analyses of the relative protein expression levels of the bands in Figure 2F. N = 3, $^{*}P < 0.05$, $^{**}P < 0.01$.

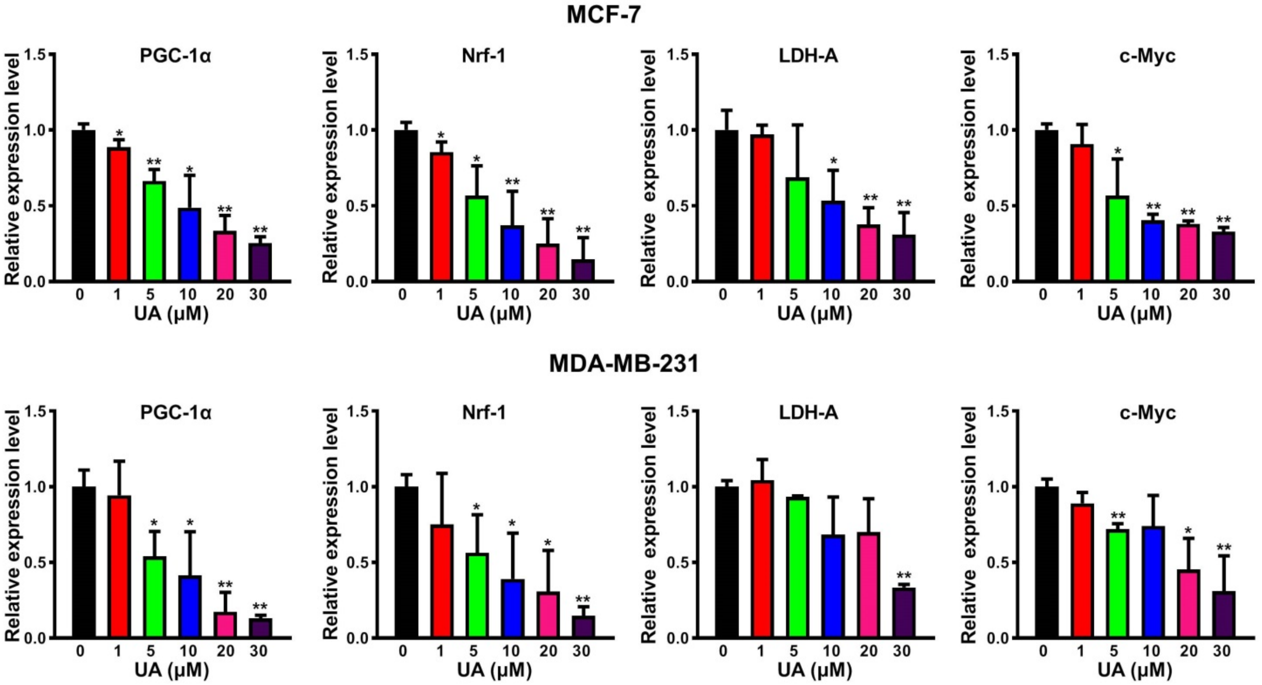

Supplementary Figure 4. Statistical analyses of the relative protein expression levels of the bands in Figure 3D. N = 3, $^{*}P < 0.05$, $^{**}P < 0.01$.

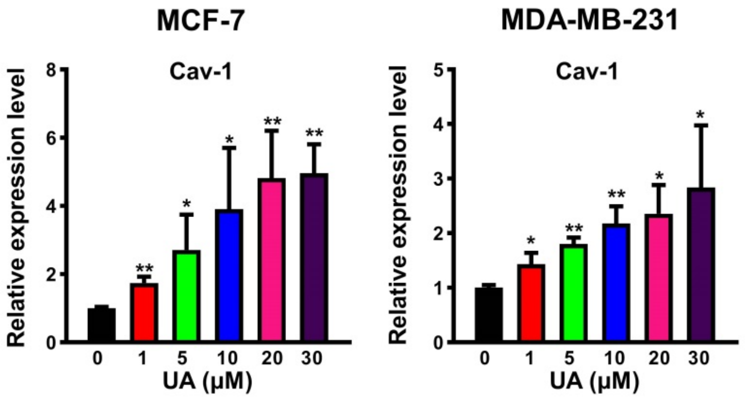

Supplementary Figure 5. Statistical analyses of the relative protein expression levels of the bands in Figure 4B. N = 3, $^{*}P < 0.05$, $^{**}P < 0.01$.

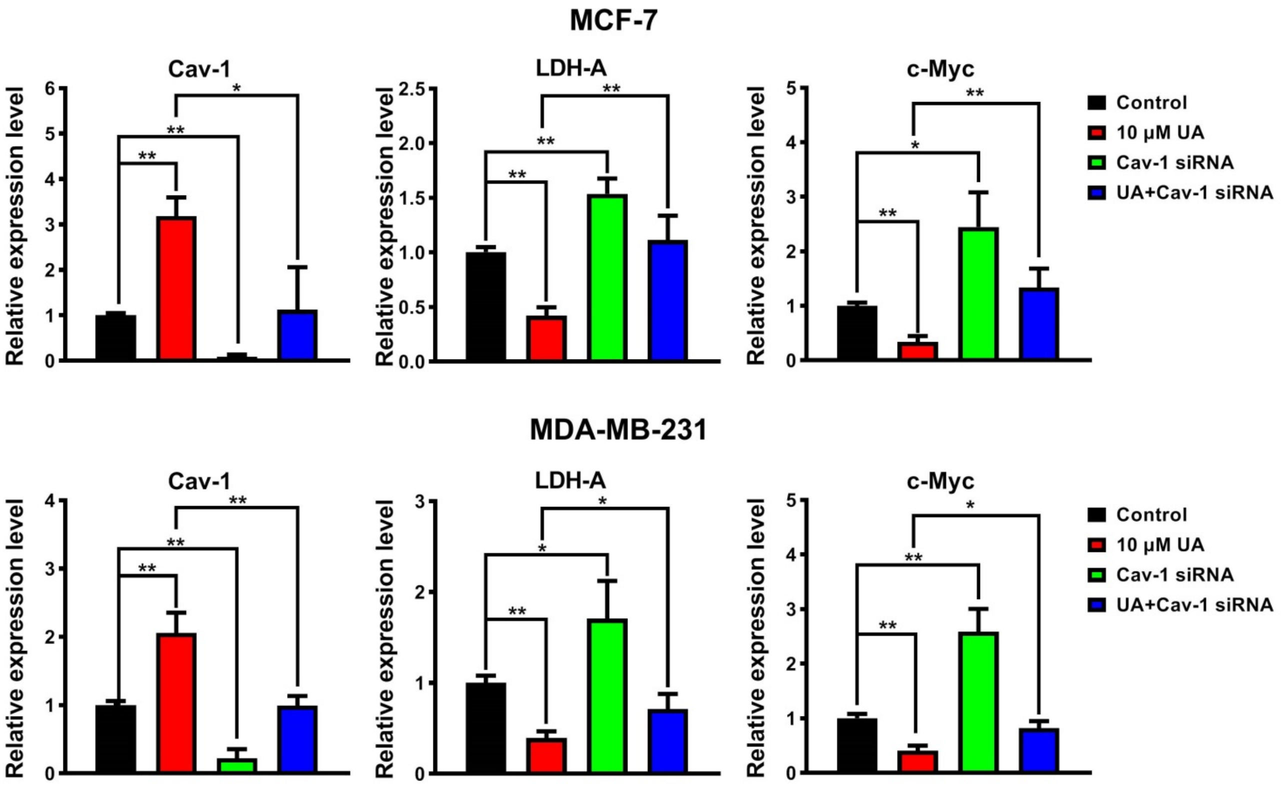

Supplementary Figure 6. Statistical analyses of the relative protein expression levels of the bands in Figure 4F. N = 3, $^{*}P < 0.05$, $^{**}P < 0.01$.

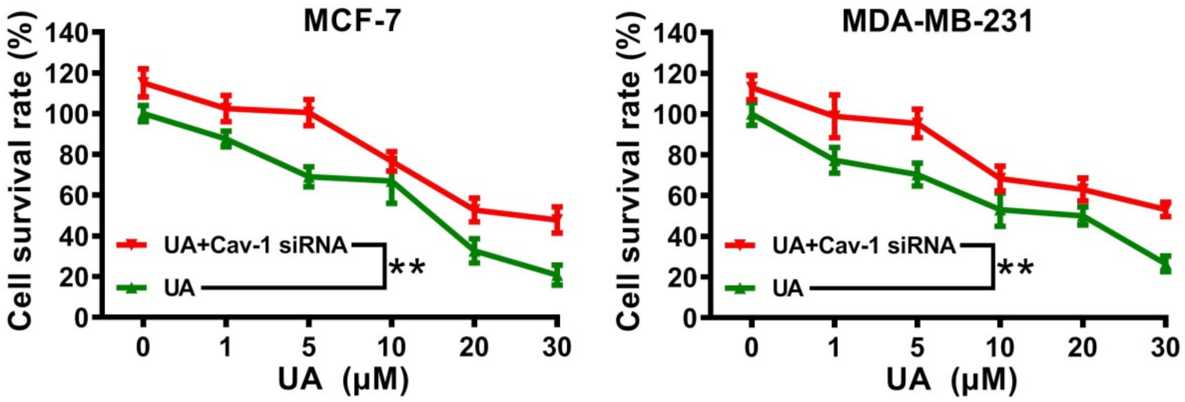

Supplementary Figure 7. The effect of Cav-1 knockdown on the growth of ursolic acid-treated breast cancer cells. MCF-7 and MDA-MB-231 cells were transfected with Cav-1 specific siRNAs and then treated with 1~30 μM ursolic acid for 48 h. Cell survival rates were detected using the CCK8 assay. N = 3, $^{**}P < 0.01$.

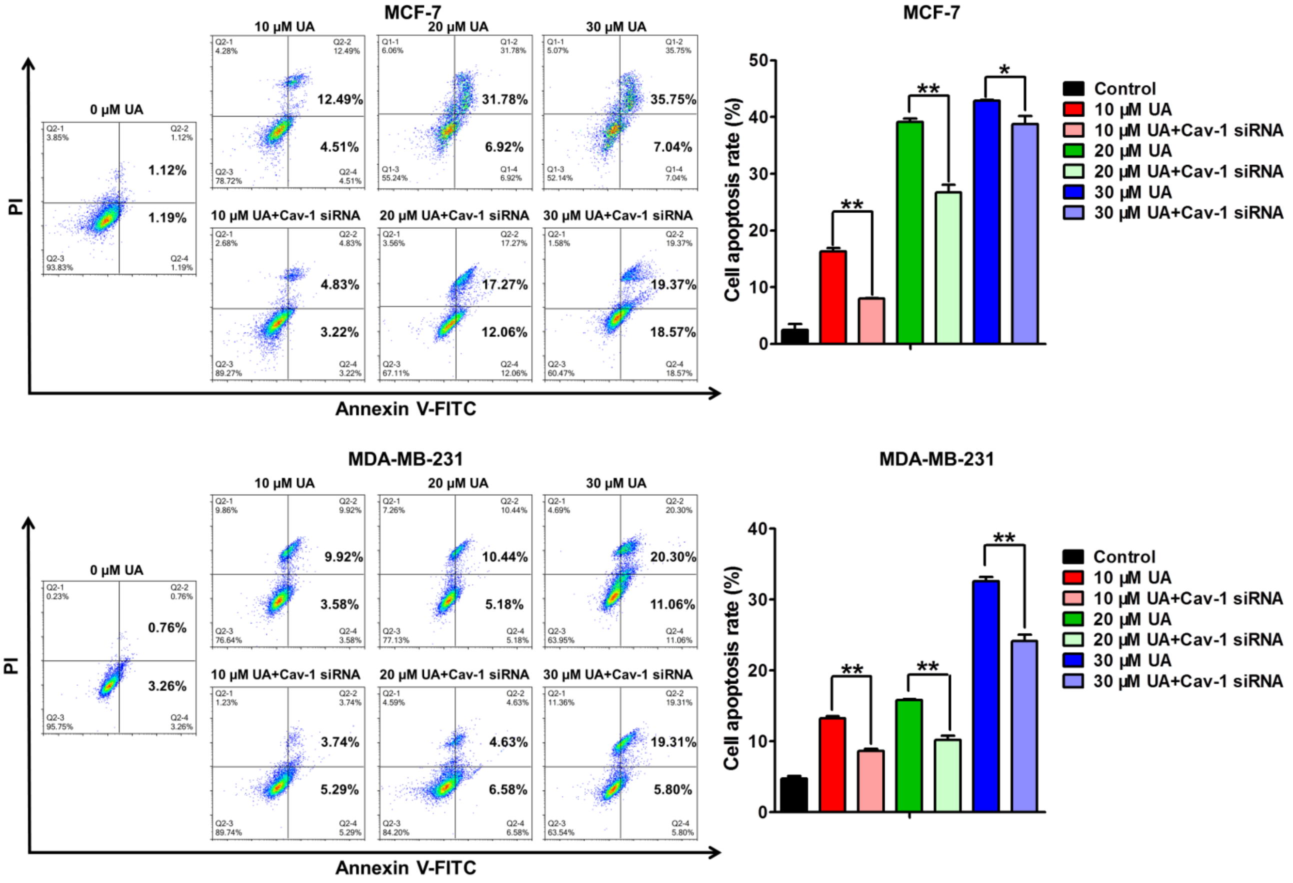

Supplementary Figure 8. The effect of Cav-1 knockdown on the apoptosis of ursolic acid-treated breast cancer cells. MCF-7 and MDA-MB-231 cells were transfected with Cav-1 specific siRNAs and then treated with 10~30 μM ursolic acid for 48 h. Cell apoptosis rates were detected using the Annexin V-FITC/PI staining assay and flow cytometry. N = 3, $^{*}P < 0.05$, $^{**}P < 0.01$.

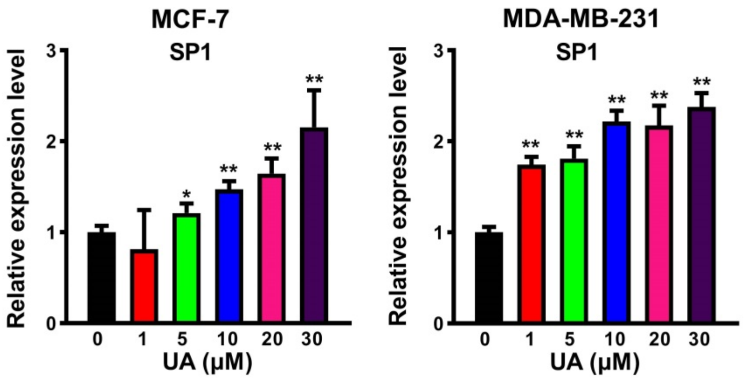

Supplementary Figure 9. Statistical analyses of the relative protein expression levels of the bands in Figure 5B. N = 3, $^{*}P < 0.05$, $^{**}P < 0.01$.

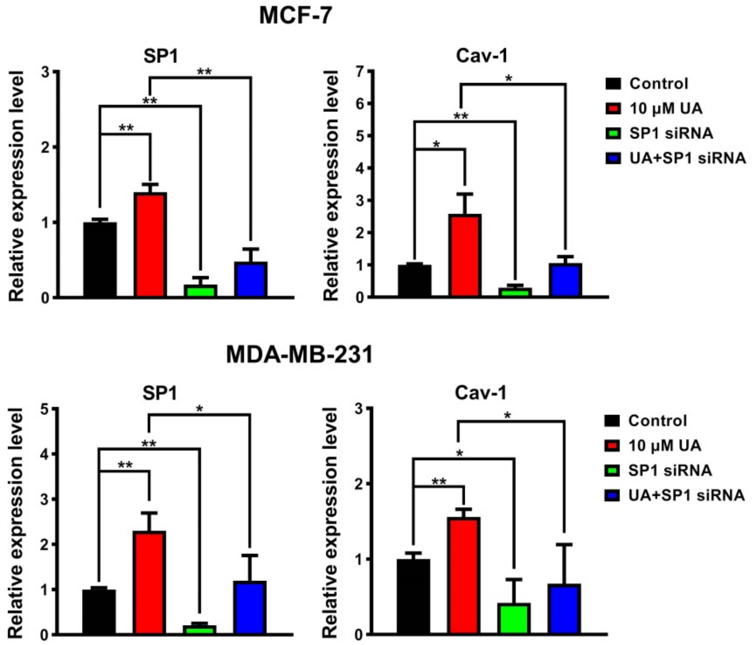

Supplementary Figure 10. Statistical analyses of the relative protein expression levels of the bands in Figure 5D. N = 3, $^{*}P < 0.05$, $^{**}P < 0.01$.

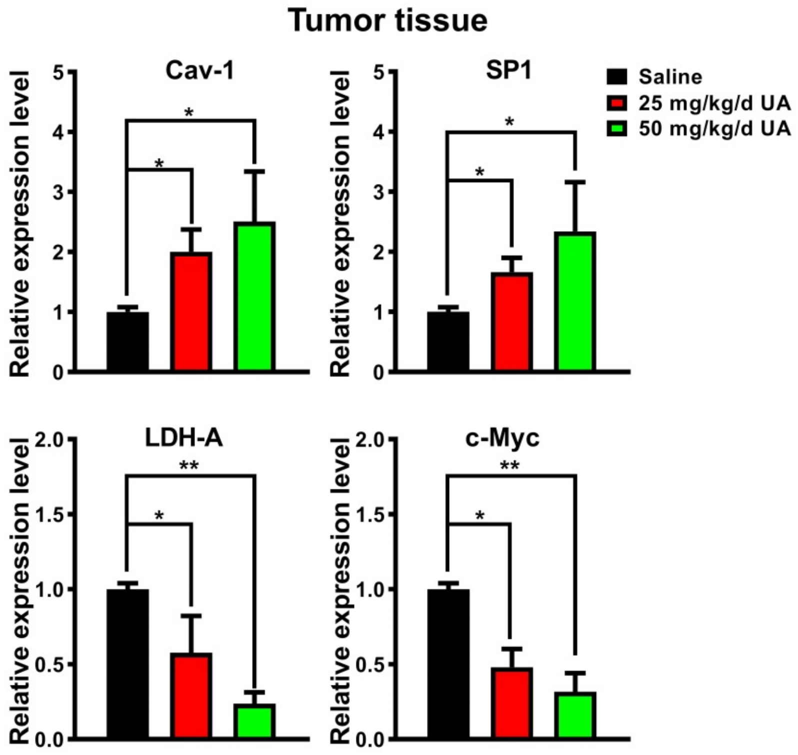

Supplementary Figure 11. Statistical analyses of the relative protein expression levels of the bands in Figure 6F. N = 3, $^{*}P < 0.05$, $^{**}P < 0.01$.
